# Healthy lifestyle behaviours and all-cause and cardiovascular mortality among 0.9 million Chinese adults

**DOI:** 10.1186/s12966-021-01234-4

**Published:** 2021-12-18

**Authors:** Xingyi Zhang, Jiapeng Lu, Chaoqun Wu, Jianlan Cui, Yue Wu, Anyi Hu, Jing Li, Xi Li

**Affiliations:** 1 National Clinical Research Center for Cardiovascular Diseases, State Key Laboratory of Cardiovascular Disease, Fuwai Hospital, National Center for Cardiovascular Diseases, Chinese Academy of Medical Sciences and Peking Union Medical College, Beijing, 100037 People’s Republic of China; 2 Health Management Center, Fuwai Hospital, National Center for Cardiovascular Diseases, Chinese Academy of Medical Sciences and Peking Union Medical College, Beijing, People’s Republic of China

**Keywords:** Healthy lifestyle behaviours, Regional distribution, Mortality, Health promotion, China

## Abstract

**Background:**

Healthy lifestyle behaviours are effective means to reduce the burden of diseases. This study was aimed to fill the knowledge gaps on the distribution, associated factors, and potential health benefits on mortality of four healthy lifestyle behaviours in China.

**Methods:**

During 2015–2019, participants aged 35–75 years from 31 provinces were recruited by the China PEACE Million Persons Project. Four healthy lifestyle behaviours were investigated in our study, including non-smoking, none or moderate alcohol use, sufficient leisure time physical activity (LTPA), and healthy diet.

**Results:**

Among 903,499 participants, 74.1% were non-smokers, 96.0% had none or moderate alcohol use, 23.6% had sufficient LTPA, 11.1% had healthy diet, and only 2.8% had all the four healthy lifestyle behaviours. The adherence varied across seven regions; the highest median of county-level adherence to all the four healthy lifestyle behaviours was in North China (3.3%) while the lowest in the Southwest (0.8%) (*p* < 0.05). Participants who were female, elder, non-farmers, urban residents, with higher income or education, hypertensive or diabetic, or with a cardiovascular disease (CVD) history were more likely to adhere to all the four healthy lifestyle behaviours (*p* < 0.001). County-level per capital Gross Domestic Product (GDP) was positively associated with sufficient LTPA (*p* < 0.05 for both rural and urban areas) and healthy diet (*p* < 0.01 for urban areas), while negatively associated with none or moderate alcohol use (p < 0.01 for rural areas). Average annual temperature was negatively associated with none or moderate alcohol use (*p* < 0.05 for rural areas) and healthy diet (*p* < 0.001 for rural areas). Those adhering to all the four healthy lifestyle behaviours had lower risks of all-cause mortality (HR 0.64 [95% CI: 0.52, 0.79]) and cardiovascular mortality (HR 0.53 [0.37, 0.76]) after a median follow-up of 2.4 years.

**Conclusions:**

Adherence to healthy lifestyle behaviours in China was far from ideal. Targeted health promotion strategies were urgently needed.

**Supplementary Information:**

The online version contains supplementary material available at 10.1186/s12966-021-01234-4.

## Background

Healthy lifestyle behaviours are considered important means to reduce the burden of diseases [1, 2]. Maintaining a healthy diet, including eating more fruit, vegetables, grains, legumes, and fish and cutting down on salt, sugar, fats, and red meat, would help avert around 11 million deaths worldwide annually [3]. Non-smoking, limited alcohol use, and sufficient leisure time physical activity (LTPA) could prevent approximately 7 million, 3 million, and 1 million deaths each year, respectively [3]. Moreover, a combination of them could yield greater health benefits, preventing over 60% of premature deaths [4] and increasing the life expectancy free of cardiovascular disease (CVD), cancer, and type 2 diabetes by 7–10 years [5].

Prior studies in China had examined the adherence to several healthy lifestyle behaviours [6–9]. However, the findings varied widely because of the discrepancies in methodology, such as inclusion criteria for participants and the definitions of healthy lifestyle behaviours. Some studies included participants whose ages were ≥ 20 years [6, 7], while other studies included merely middle-aged adults [8, 9]. With respect to the measurement of physical activity, some studies calculated the total metabolic equivalent tasks (METs) for all types of activities (occupational, commuting, domestic, and leisure-time) and defined the higher half as the healthy group [8, 9], while other studies only included the leisure time physical activities, and made the definition of “healthy” based on guidelines [7]. The definitions of healthy diet were more complicated. Most of the studies merely asked about eating frequencies [8, 9], while only a few ones collected information on the consumption of each food group and made the definitions based on it [6, 7]. In addition to the conventional food such as fruit, vegetables, and red meat, some studies also took sodium intake or consumption of tea into consideration [6, 7]. The association between healthy lifestyle behaviours and risk of all-cause and cardiovascular mortality had been evaluated in some large cohorts, like China Kadoorie Biobank (CKB) study [3, 9, 10]. Nevertheless, as most of the studies were limited by incomplete geographical coverage, whether the adherence varied by population subgroups or regions is still unknown, and few have investigated the regional economic or environmental factors related to adherence to healthy lifestyle behaviours [11]. These evidences are essential for developing and implementing targeted health promotion strategies that are needed in China.

To fill these knowledge gaps, we reported the findings from China Patient-centered Evaluative Assessment of Cardiac Events (PEACE) Million Persons Project (MPP), which is a population-based screening project covering all 31 provinces in mainland China. We aimed to examine the distribution of four healthy lifestyle behaviours among Chinese adults, evaluate the degree of clustering of these lifestyle behaviours, identify individual and regional factors associated with the adherence, and investigate its association with all-cause and cardiovascular mortality.

## Methods

### Study design and population

The China PEACE MPP is a public health project on screening and management of high-risk subjects with CVD funded by Chinese government. The study design has been previously published [12]. Briefly, the project sampled 252 sites (152 rural counties, 100 urban districts) in 31 provinces (around 8 for each) from September 2015 to November 2019 (Additional file 1: Section 1 and Fig. S1). Residents living in the region for at least 6 months in the prior year, aged 35 to 75 years, were approached as participants. Enrolled participants with serial project ID number ending with 1, 3, 5, or 7 were randomly sampled as representatives of the entire cohort to provide detailed information of CVD. The project protocol was approved by the central ethics committee at Fuwai Hospital, Beijing, China.

We excluded participants who had missing information for lifestyle behaviours (*n* = 77,543) or demographic data (*n* = 2434). When demonstrating the county-level distributions of adherence, we excluded 18 counties with less than 1000 eligible participants enrolled (*n* = 11,056). We excluded participants who reported previous medical history of CVD in Cox regression analysis (*n* = 34,832) (Additional file 1: Fig. S2 and Table S1).

### Data collection and variables

Data of four lifestyle factors, namely, smoking, alcohol use, LTPA, and diet, was collected through standardized in-person questionnaire interviews, which was similar to other large-scale population-based studies [13, 14]. Briefly, we inquired about smoking status (never, former, or current smokers); ever smokers were further asked about the frequency and type of cigarette smoking as well as the amount of tobacco consumed per day, and former smokers were additionally asked about the reasons for cessation. For alcohol use, we asked about drinking frequency (‘never’, ‘once or less per month’, ‘2-4 times per month’, ‘2-3 times per week’, ‘more than 4 times per week’). Ever drinkers were asked about the amount of alcohol consumption during a typical drinking day. On the basis of these information, an average daily alcohol consumption was estimated. LTPA was quantified by the typical types of activity at different intensity (swimming, running or aerobic exercise as vigorous- intensity activity; ball games/walking/gymnastics/folk dancing/Tai-Chi/qigong or other exercise as moderate-intensity activity), frequency, and exercise time per week. A qualitative food frequency questionnaire was used to collect habitual dietary intake by asking about eating frequency of 12 food groups during the past year. For each food group, five frequency categories were provided (‘daily’, ‘4-6 days per week’, ‘1-3 days per week’, ‘1-3 days per month’, ‘never or almost never’) (Additional file 1: Section 2). A customized electronic data collection system with real-time logical check function was used to ensure the quality and completeness of interview data.

The healthy group regarding smoking was defined as never smokers or former smokers who stopped by choice as recommended [15]. The healthy group for alcohol use was defined as never drinkers, or drinkers who drank no more than 25 g (for male) or 15 g (for female) per day on average, according to the Chinese dietary guideline [16]. Participants who performed at least 150 min of moderate-intensity aerobic activities or 75 min of vigorous-intensity aerobic activities per week were considered as having sufficient LTPA, which was regarded as healthy for this study [17]. Based on the recommendations in the Chinese Dietary Guidelines [16], a healthy diet score was calculated by the weekly intake of 6 food groups, including the daily intake of fresh fruit, fresh vegetables, and whole grains, and eating fish and other seafood ≥1 day per week, bean and bean food ≥4 days per week, and red meat < 7 days per week [8, 9]. For each food group, the participant who met the criterion scored 1, otherwise, scored 0. The full mark of the healthy diet score was 6 and the healthy group was defined as those whose score ≥ 4 (Additional file 1: Section 2).

Participants’ demographic and socio-economic characteristics (i.e., gender, age, occupation, education, household income, marriage, and social medical insurance), urbanicity, and medical history (i.e., self-reported hypertension or diabetes and CVD history [myocardial infarction or stroke]) were collected through the questionnaire interview. Per capital Gross Domestic Product (GDP) and average annual temperature in 2017, which reflected socio-economic and environmental characteristics of the counties, were obtained from grey literature including statistics yearbooks. Seven geographical regions, including Northeast, North China, East China, Central China, South China, Northwest, and Southwest, were classified by official geographical divisions of China (Additional file 1: Fig. S1).

### Ascertainment of outcomes

We ascertained the vital status of participants through the National Mortality Surveillance System and Vital Registration of Chinese Center for Disease Control and Prevention (CDC). All events were coded using International Classification of Diseases (ICD)-10. The outcomes of interest in this study were all-cause and cardiovascular mortality (ICD-10: I01-I99). When analyses were conducted, mortality data were available up to 31 December 2019. Therefore, we censored the follow-up at this date or the date of death, whichever occurred first.

### Statistical analysis

We described the characteristics and distribution of four healthy lifestyle behaviours among all participants. Categorical variables were summarized as frequencies and percentages, and continuous variables as means ± standard deviations or medians [interquartile range (IQR)]. Standardized mean differences (SMD) was computed to compare the differences between male and female, as well as between rural and urban areas. When the absolute value of the SMD is < 0.2, the difference is considered “small” [18]. The Mantel-Haenszel test for trends and simple linear regression were used to detect the linear trends.

If a combination of two or more lifestyle behaviours is more prevalent than it can be expected on the basis of the prevalence of each lifestyle behaviour, it is called “clustering”. To evaluate the clustering of multiple healthy lifestyle behaviours, the ratio between the observed and expected (O/E) adherence was calculated for each possible combination. The observed adherence was identified in the study population. The expected adherence was computed by multiplying the separate probabilities of each lifestyle on the basis of their occurrence. It was assumed that the healthy lifestyle behaviours occurred independently. Hence, we could identify the clustering where O/E ratio was above 1 [19].

We constructed heat maps of the adherence to four healthy lifestyle behaviours, and the rates of adherence to healthy lifestyle behaviours had been standardized by age and gender structure from the 2010 population census of China. We included 234 counties after excluding those with less than 1000 eligible participants. Based on the data on adherence in these counties, we estimated the other 2660 non-study counties in mainland China using interpolation models of inverse distance weighing, which was the inversed squared distances weighted average of neighbouring counties [20]. We filled the polygons of counties with scaled colours according to their adherence. Kruskal-Wallis tests were used to test for the differences of county-level adherence among seven geographical regions. The median odds ratio (MOR) [21] at county-level was calculated as well to describe the regional variation by applying multilevel models with random effect, which indicates the median value of the odds ratios obtained when comparing the odds of the occurrence of the outcome in an individual from a randomly selected cluster with another individual with identical covariates but from a different randomly selected cluster when the clusters are ordered by risk [21].

Multivariable logistic regression models were adopted to investigate individual characteristics associated with adherence to four healthy lifestyle behaviours, comprising demographic and socio-economic characteristics (i.e., gender, age, occupation, education, household income, marriage, and social medical insurance), urbanicity, and medical history (i.e., self-reported hypertension or diabetes and CVD history [myocardial infarction or stroke]). For each healthy lifestyle behaviour that was assessed, we adjusted for other three lifestyles simultaneously in the model. Scatter plots and fitting lines were used to demonstrate the correlations of four healthy lifestyle behaviours with regional characteristics (i.e., per capital GDP and average annual temperature at county level). The heterogeneity of correlation coefficients between rural and urban areas were tested by Fisher’s z transformation.

Cox proportional hazard models (crude, adjusting for age and gender, or multivariable adjusted models) were used to calculate hazard ratio (HR) and 95% confidence interval (CI) for the healthy lifestyle behaviours with all-cause mortality and cardiovascular mortality. Multivariable adjusted models included most of the demographic and socioeconomic characteristics, i.e., gender, age, occupation, education, household income, marriage, social medical insurance, urbanicity, region, and county level per capital GDP. We conducted a sensitivity analysis by excluding deaths that occurred within the first six months. We conducted another sensitivity analysis by excluding those with self-reported hypertension or diabetes.

All analyses were conducted with SAS 9.4 (SAS Institute Inc., Cary, North Carolina) and R 3.6.2 (The R Foundation for Statistical Computing, Vienna, Austria).

## Results

### Participant characteristics and healthy lifestyle behaviours

Among the 903,499 participants included, the mean age was 55.9 ± 9.8 years and 60.6% were female. Overall, 48.9% of participants were farmers, 61.3% were living in rural areas, 22.5% had received high school education or above, 17.6% had an annual household income over 50,000 Yuan, 92.9% were currently married, and 98.3% had social medical insurance. Among all participants, 28.0% had self-reported hypertension or diabetes, and 3.9% had a history of CVD (Table 1).

**Table 1:** Participant characteristics by number of healthy lifestyle behaviours

	Total	Number of healthy lifestyle behaviours^a^					
		0	1	2	3	4	P for trend
	903,499	13,787	128,887	513,275	216,846	30,704	
**Demographic and socio-economic**							
Female	547,599 (60.6)	435 (3.2)	10,300 (8.0)	353,431 (68.9)	159,993 (73.8)	23,440 (76.3)	< 0.001
Age, year	55.9 ± 9.8	55.5 ± 9.1	56.0 ± 9.7	55.4 ± 9.9	56.8 ± 9.7	57.8 ± 9.6	< 0.001
Occupation: Farmer	442,244 (48.9)	8748 (63.5)	79,611 (61.8)	282,559 (55.1)	66,315 (30.6)	5011 (16.3)	< 0.001
Education: high school or above	202,961 (22.5)	2215 (16.1)	23,346 (18.1)	95,689 (18.6)	68,422 (31.6)	13,289 (43.3)	< 0.001
Household income: 50,000 yuan per year or above	158,669 (17.6)	2210 (16.0)	19,126 (14.8)	78,146 (15.2)	49,454 (22.8)	9733 (31.7)	< 0.001
Marriage	839,653 (92.9)	12,982 (94.2)	120,974 (93.9)	477,977 (93.1)	199,437 (92.0)	28,283 (92.1)	< 0.001
Social medical insurance	887,809 (98.3)	13,582 (98.5)	126,824 (98.4)	504,519 (98.3)	212,829 (98.1)	30,055 (97.9)	0.982
**Medical history**							
Hypertension or diabetes	253,317 (28.0)	3745 (27.2)	33,333 (25.9)	133,366 (26.0)	71,881 (33.1)	10,992 (35.8)	< 0.001
CVD history	34,832 (3.9)	334 (2.4)	4931 (3.8)	17,323 (3.4)	10,550 (4.9)	1694 (5.5)	< 0.001
**Regional characteristics**							
Urbanicity: rural	553,587 (61.3)	10,010 (72.6)	91,937 (71.3)	338,426 (65.9)	103,388 (47.7)	9826 (32.0)	< 0.001

^a^ Healthy lifestyle behaviours were defined as non-smoking, none or moderate alcohol use, sufficient leisure time physical activity, and healthy diet

After standardizing age and gender using the 2010 national census data, none or moderate alcohol use had the highest adherence (96.0%), followed by non-smoking (74.1%), while the adherence to sufficient LTPA (23.6%) and healthy diet (11.1%) were relatively low (Table 2). Only 2.8% of all participants adhered to all the four heathy lifestyle behaviours. More people than expected adhered to all (O/E ratio = 1.5) or none (O/E ratio = 3.4) of the four healthy lifestyle behaviours. The most common pair of healthy lifestyle behaviours was non-smoking and none or moderate alcohol use, with the highest observed adherence of 51.3%. The most notable clustering pattern was sufficient LTPA and healthy diet, the adherence to which was 2.9 times higher than expected (O/E ratio = 2.9) (Additional file 1: Table S2).

**Table 2:** Participants’ adherence to healthy lifestyle behaviours, overall, by gender and by urbanicity

	Overall	Female	Male	SMD	Urban	Rural	SMD
	903,499	547,599	355,900		349,912	553,587	
Non-smoking	716,604 (74.1)	536,568 (98.2)	180,036 (50.9)	1.295	285,143 (76.2)	431,461 (72.9)	0.075
None or moderate alcohol use	873,146 (96.0)	542,511 (99.2)	330,635 (92.9)	0.329	339,909 (96.5)	533,237 (95.7)	0.039
Sufficient LTPA	238,072 (23.6)	147,480 (24.4)	90,592 (22.9)	0.035	129,765 (31.9)	108,307 (18.4)	0.314
Healthy diet	100,969 (11.1)	64,342 (11.8)	36,627 (10.4)	0.047	55,717 (15.5)	45,252 (8.3)	0.223
≥1 heathy lifestyle behaviours	889,712 (98.0)	547,164 (99.9)	342,548 (96.2)	0.272	346,135 (98.5)	543,577 (97.8)	0.054
≥2 heathy lifestyle behaviours	760,825 (80.0)	536,864 (98.3)	223,961 (62.2)	1.015	309,185 (84.0)	451,640 (77.4)	0.168
≥3 heathy lifestyle behaviours	247,550 (24.0)	183,433 (31.6)	64,117 (16.7)	0.352	134,336 (32.7)	113,214 (18.5)	0.329
4 heathy lifestyle behaviours	30,704 (2.8)	23,440 (3.9)	7264 (1.8)	0.125	20,878 (4.8)	9826 (1.6)	0.180

The frequencies in the table were based on rough counting numbers before standardization, while the percentages were standardized by age and gender using the 2010 national census data

### Variations in the adherence to healthy lifestyle behaviours

Compared with men, women had higher adherence to non-smoking and none or moderate alcohol use (both SMD > 0.2). On sufficient LTPA and healthy diet, urban residents’ adherence after standardization was higher than their rural counterparts’ (both SMD > 0.2) (Table 2). Regarding the combined healthy lifestyle behaviours, women had a higher standardized adherence (98.3%) to two or more healthy lifestyle behaviours compared with men (62.2%) (SMD > 0.2), and urban residents had a higher standardized adherence to three or more healthy lifestyle behaviours (32.7%) than their rural counterparts (18.5%) (SMD > 0.2) (Table 2, Additional file 1: Fig. S3).

**Fig. 1:**
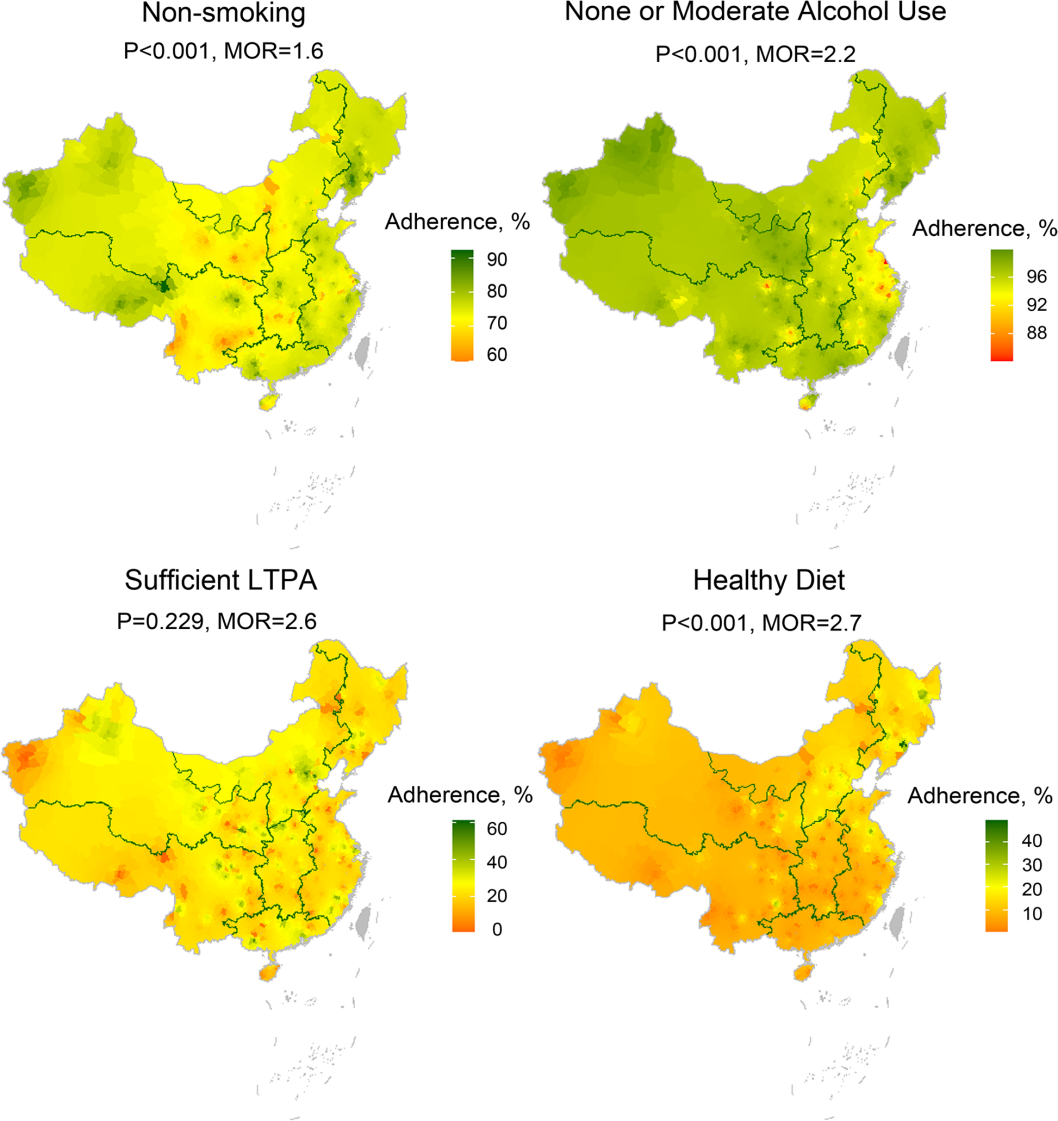
Adherence to four healthy lifestyle behaviours in China. LTPA: leisure time physical activity; P values: P values for Kruskal-Wallis tests for the differences of county-level adherence among seven geographical regions

The standardized adherence for all the four healthy lifestyle behaviours ranged from 0.0–14.9% among the counties/districts with an MOR of 3.4. The highest median of county-level adherence to all the four healthy lifestyle behaviours was in North China (3.3%) while the lowest in the Southwest (0.8%) (*p* < 0.05) (Additional file 1: Table S3). The geographical distributions of adherence to the four individual healthy lifestyle behaviours were different. The Northwest, North China, Central China, and the Southwest had the lowest adherence in non-smoking, East China had the lowest adherence in none or moderate alcohol use, the Northeast had the lowest adherence in sufficient LTPA, and the Southwest had the lowest adherence in healthy diet (Fig. 1, Additional file 1: Table S4).

### Factors associated with four healthy lifestyle behaviours

**Fig. 2:**
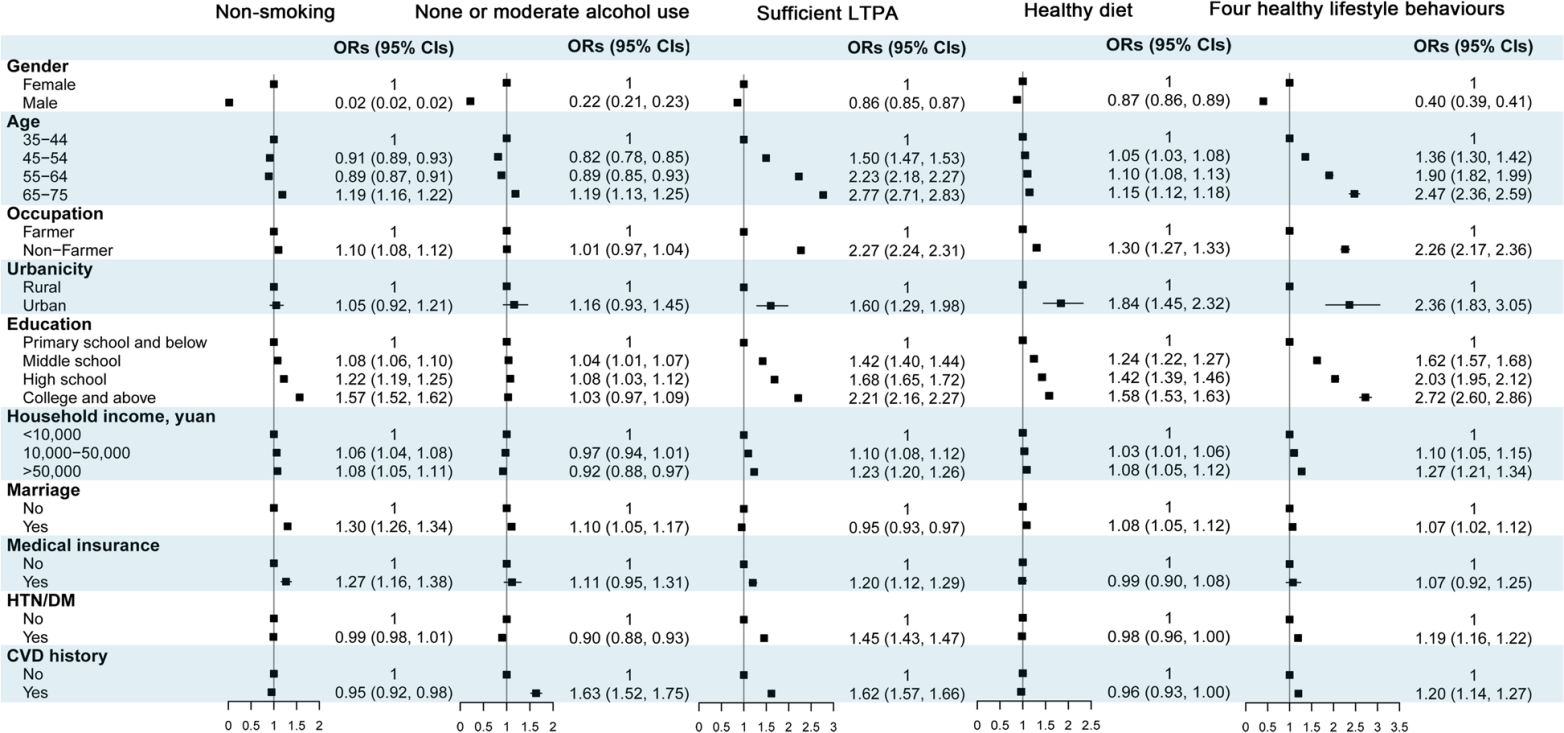
Individual characteristics associated with adherence to four healthy lifestyle behaviours

Multivariable logistic regression analysis showed that the individual characteristics were associated with four healthy lifestyles in different ways. Higher household income was negatively associated with none or moderate alcohol use, although positively associated with adherence to the other three healthy lifestyle behaviours (Fig. 2). In general, participants who were female, elder, non-farmers, living in urban areas, with higher education or higher household income, hypertensive or diabetic, or with a CVD history were more likely to adhere to all the four healthy lifestyle behaviours (*p* < 0.001) (Fig. 2).

**Fig. 3:**
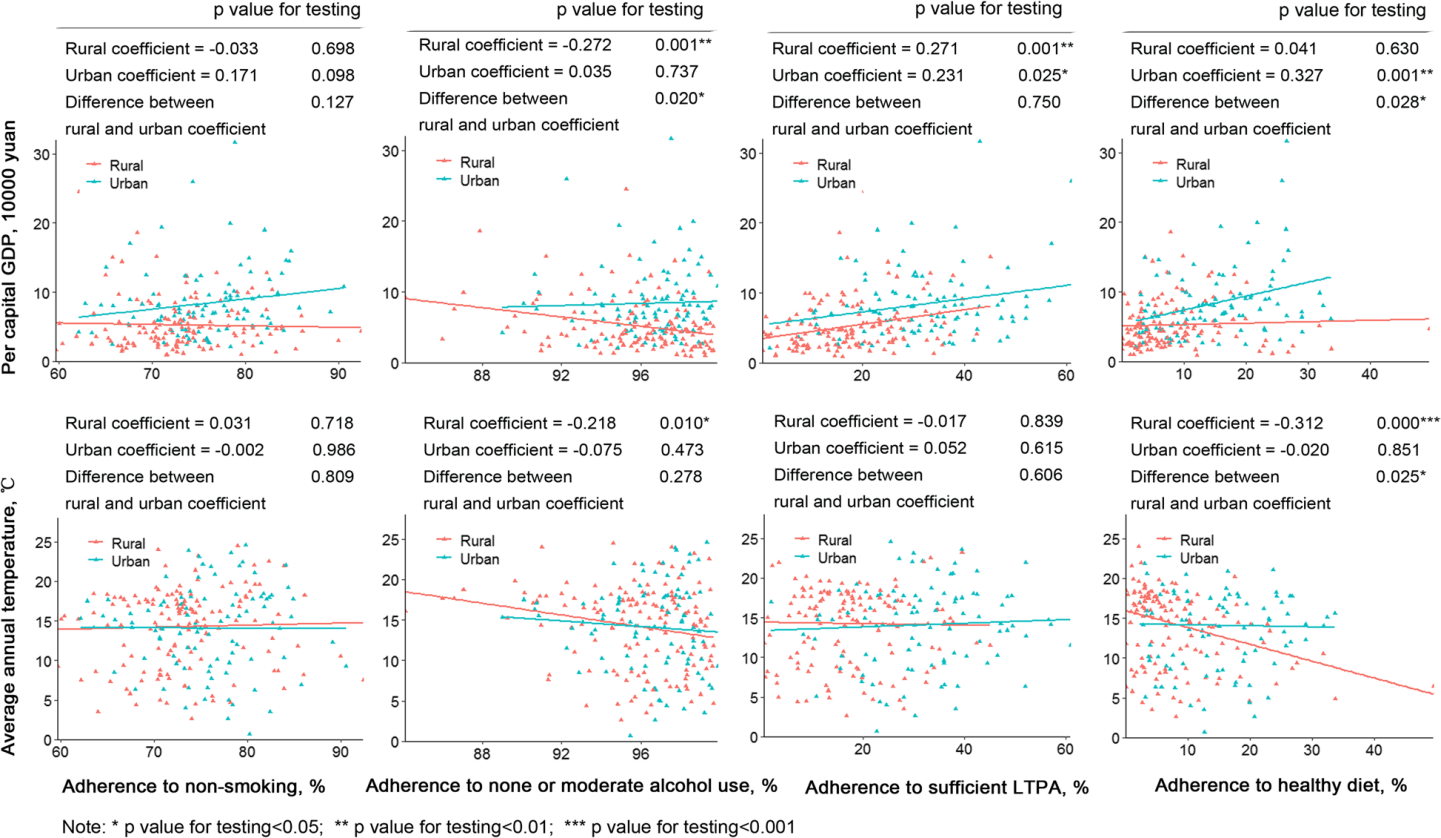
Correlations of the adherence to four healthy lifestyle behaviours with regional socio-economic and environmental characteristics

Among the counties/districts, per capital GDP was positively associated with sufficient LTPA (*p* < 0.05 for both rural and urban areas) and healthy diet (*p* < 0.01 for urban areas), while negatively associated with none or moderate alcohol use (p < 0.01 for rural areas). Average annual temperature was negatively associated with none or moderate alcohol use (p < 0.05 for rural areas) and healthy diet (p < 0.001 for rural areas) (Fig. 3). In general, higher adherence to all the four healthy lifestyle behaviours was significantly associated with higher per capital GDP (p < 0.05 for both rural and urban areas), while negatively associated with average annual temperature (p < 0.05 for rural areas) (Additional file 1: Fig. S4).

### Health outcomes associated with four healthy lifestyle behaviours

A total of 868,667 participants were included in the analysis. The median follow-up time was 2.4 years (IQR: 1.3–3.6 years). There were 9156 deaths (1.05%, mortality rate 4.65 [4.56–4.75]/1000 person-years) and 3474 CVD deaths (0.40%, mortality rate 1.77 [1.71–1.83]/1000 person-years) in all the participants included. When the healthy lifestyle behaviours were combined, the number of healthy lifestyle behaviours showed significant inverse linear relationships with the risks of all-cause and cardiovascular mortality (all p for trend< 0.001). Compared with the participants without any healthy lifestyle behaviours, the multivariable adjusted hazard ratio of participants who adhered to all the four healthy lifestyle behaviours was 0.64 [95% confidence interval (CI): 0.52, 0.79] for all-cause mortality and 0.53 [0.37, 0.76] for cardiovascular mortality, respectively (Table 3).

**Table 3:** Hazard ratios for all-cause and cardiovascular mortality

	All-cause mortality		Cardiovascular mortality	
	HR (95% CI)	***P*** value	HR (95% CI)	P value
**Model 1: no adjustment**				
Number of healthy lifestyle behaviours (reference = 0)				
1	1.02 (0.89, 1.18)	0.766	1.13 (0.89, 1.44)	0.321
2	0.68 (0.60, 0.78)	< 0.001	0.80 (0.63, 1.01)	0.063
3	0.56 (0.49, 0.64)	< 0.001	0.68 (0.53, 0.86)	0.002
4	0.47 (0.39, 0.57)	< 0.001	0.53 (0.38, 0.74)	< 0.001
Non-smoking	0.61 (0.58, 0.64)	< 0.001	0.66 (0.61, 0.71)	< 0.001
None or moderate alcohol use	0.88 (0.79, 0.98)	0.019	0.98 (0.82, 1.18)	0.855
Sufficient LTPA	0.84 (0.80, 0.88)	< 0.001	0.84 (0.77, 0.91)	< 0.001
Healthy diet	0.81 (0.75, 0.87)	< 0.001	0.83 (0.74, 0.94)	0.002
**Model 2: adjusted for age and gender**				
Number of healthy lifestyle behaviours (reference = 0)				
1	0.98 (0.85, 1.13)	0.776	1.07 (0.83, 1.36)	0.612
2	0.87 (0.76, 1.00)	0.051	0.94 (0.74, 1.20)	0.619
3	0.66 (0.57, 0.76)	< 0.001	0.72 (0.56, 0.93)	0.011
4	0.52 (0.43, 0.64)	< 0.001	0.53 (0.38, 0.74)	< 0.001
Non-smoking	0.84 (0.80, 0.89)	< 0.001	0.85 (0.78, 0.93)	< 0.001
None or moderate alcohol use	1.07 (0.96, 1.19)	0.214	1.13 (0.94, 1.35)	0.184
Sufficient LTPA	0.72 (0.69, 0.76)	< 0.001	0.71 (0.65, 0.77)	< 0.001
Healthy diet	0.82 (0.76, 0.88)	< 0.001	0.84 (0.75, 0.94)	0.003
**Model 3: multivariable adjusted model**				
Number of healthy lifestyle behaviours (reference = 0)				
1	0.94 (0.81, 1.09)	0.445	0.97 (0.75, 1.26)	0.842
2	0.89 (0.77, 1.03)	0.111	0.91 (0.70, 1.17)	0.452
3	0.74 (0.63, 0.86)	< 0.001	0.75 (0.58, 0.98)	0.035
4	0.64 (0.52, 0.79)	< 0.001	0.53 (0.37, 0.76)	0.001
Non-smoking	0.88 (0.83, 0.93)	< 0.001	0.90 (0.82, 0.99)	0.024
None or moderate alcohol use	1.08 (0.97, 1.21)	0.156	1.05 (0.87, 1.27)	0.587
Sufficient LTPA	0.82 (0.78, 0.87)	< 0.001	0.77 (0.71, 0.85)	< 0.001
Healthy diet	0.90 (0.83, 0.97)	0.008	0.89 (0.78, 1.01)	0.070

Model 3: adjusted for gender, age, occupation, education, household income, marriage, social medical insurance, urbanicity, region, and county level per capital gross domestic product (GDP)

For individual healthy lifestyle behaviours that were used to fit multivariable adjusted Cox regression models separately, lower risks of all-cause mortality were observed in non-smoking (HR: 0.88 [0.83, 0.93], sufficient LTPA (HR: 0.82 [0.78, 0.87]) and healthy diet (HR: 0.90 [0.83, 0.97] (all *P* < 0.01), but not found in none or moderate alcohol use (HR: 1.08 [0.97, 1.21]). The adjusted HRs for cardiovascular mortality were almost the same as those for all-cause mortality. Similar findings were identified in the sensitivity analyses excluding the deaths that occurred within the first six months of follow-up (Additional file 1: Table S5) or excluding those with self-reported hypertension or diabetes (Additional file 1: Table S6).

## Discussion

In this study, we found that only 1 in 35 adults in China adhered to the four heathy lifestyle behaviours, i.e., non-smoking, none or moderate alcohol use, sufficient LTPA, and healthy diet. More people than expected adhered to all or none of the four healthy lifestyle behaviours. The adherence varied across seven regions, with the highest adherence in North China and the lowest in the Southwest. Individuals who were female, elder, of higher socio-economic status (SES), hypertensive or diabetic, or having a CVD history were more likely to adhere to all the four healthy lifestyle behaviours. At the regional level, GDP and temperature were significantly correlated with the adherence. Adherence to all the four healthy lifestyle behaviours was associated with a 40% reduction in all-cause deaths and a 50% reduction in cardiovascular deaths.

Previous studies have reported suboptimal adherence to healthy lifestyle behaviours in Chinese population [6, 22], and CKB identified that the total mortality risk was considerably lower for participants who adhered to a combination of multiple healthy behaviours [9]. In addition to confirming these findings, our study extends the existing literature in several aspects. First, the study depicted the distribution of adherence to healthy lifestyle behaviours across the population and identified the groups with larger gaps. In general, people of low SES, such as those living in rural areas or having low levels of income or education, tended to have lower adherence to healthy lifestyle behaviours. This may be attributed to their poor health literacy [23]. However, adherence to none or moderate alcohol use was negatively correlated with income levels. This may be due to the fact that people of higher SES are more frequently exposed to alcohol out of work or social needs [24].

Second, this study comprehensively demonstrated regional variations of adherence to healthy lifestyle behaviours in China and revealed its correlation to major environmental and socio-economic indicators. On the one hand, the variance in overall adherence across regions was notable, indicating that more attention should be attached to the regions with large gaps, such as the Southwest. On the other hand, the adherence to four individual healthy lifestyle behaviours varied widely across regions as well. It could be directly related to the local burden of certain diseases. For example, lower adherence to healthy diet in the South China potentially lead to the higher prevalence of prediabetes in these areas [25]. The positive correlation between per capital GDP and adherence to most of the healthy lifestyle behaviours hinted at the promoting effects of socio-economic development on health. The higher level of health literacy in more developed regions could be the reason behind it [26, 27]. Geographic agglomeration of agriculture products related to temperature (e.g. more corn, beans, and livestock in the north) may explain the association between temperature and the observed adherence to healthy diet [28, 29].

Prior studies demonstrated that in western population, a combination of four or more healthy lifestyle behaviours was related to a 66% reduced risk of all-cause mortality [3], meanwhile the potential health benefits in Chinese population seemed similar [9]. However, limited alcohol intake was not found to have a prominent association with all-cause mortality in our study, which was different from the results of CKB [9]. The negative association may be due to the short follow-up time, or caused by potential reverse causality. Nevertheless, the positive associations between multiple healthy lifestyle behaviours and health outcomes were overwhelming. The inverse relationship between the number of healthy lifestyle behaviours and mortality risks was significant. This is consistent with previous studies [9, 30, 31], and can be related to the clustering of healthy lifestyle behaviours. Similar results were seen in the UK biobank, where a combined weighted lifestyle score was developed and used to categorise the participants into very unhealthy, unhealthy, healthy or very healthy. A very healthy lifestyle score was associated with 6.3 years of longer life expectancy for men and 7.6 years for women, regardless of the presence of multimorbidity [10].

Our findings have some policy implications for chronic disease prevention and control. First, particular attention is needed for the vulnerable groups (i.e. those with lower education and income levels, or living in rural areas), in addition to the health promotion projects for the general population, like the *China Healthy Lifestyle behaviours for All* [32]. Second, in a large country like China, health promotion strategies should be tailored to the varied regional conditions; meanwhile, focused improvements in major local health issues would be more efficient. Third, the fact that many people are not engaging in sufficient physical activity or eating a diet in accordance with the Chinese Dietary Guidelines suggests that integrated interventions, such as consultations on both diet and exercise, could be a more practical way to maximize the impact of health promotion on improvement of adherence to healthy lifestyle behaviours in the general population.

The study was subject to some limitations as well. First of all, some recall biases and measurement errors were inevitable since the lifestyle factors were self-reported; even we used the standardized questionnaire. We did not conduct validation for the questionnaires of physical activity or diet in this study; however, the questions had been used and validated in other large population studies [13, 14]. The food groups were only measured in frequency (not portion size) and there was no adjustment for the overall energy intakes. Secondly, reverse causation and residual confounding due to unmeasured or unknown factors cannot be completely ruled out, although we have included most of the demographic and socioeconomic characteristics in the multivariable models. Third, although the National Mortality Surveillance System and Vital Registration cover urban and rural areas in all of the 31 provinces of mainland China and missing death records will be registered when they are validated by local Bureau of Household Registration, there might still be a very small proportion of missing death records particularly in the remote rural areas. And study participants were followed up for a relatively short period. These may lead to a slight underestimation of the potential impact of adherence to healthy lifestyle behaviours on mortality. Fourth, the study population was not established based on a random sampling design, which potentially prohibited estimation of national or regional averages, and may cause spurious associations [33]. In the current study, we only included four lifestyle behaviours; other lifestyle behaviours, such as sedentary behaviour or sleep deprivation, also require in-depth investigation in future studies.

## Conclusions

In conclusion, the adherence to healthy lifestyle behaviours in China was far from ideal; it varied substantially across population subgroups and geographical regions. Targeted health education and health promotion strategies should be elevated to a national public policy priority.

## Supplementary Information

**Additional file 1.** Supplementary material and tables.

## Data Availability

The authors declare that all data supporting the findings of this study are available within the article and its supplementary information files.

## Abbreviations

CVD: Cardiovascular diseases
LTPA: Leisure time physical activity
China-PAR: Prediction for Atherosclerotic Cardiovascular Disease Risk in China
China PEACE MPP: China Patient-centered Evaluative Assessment of Cardiac Events Million Persons Project
NCCD: National Center for Cardiovascular Disease, Beijing, China
GDP: Gross Domestic Product
CDC: Center for Disease Control and Prevention
ICD-10: International Classification of Diseases 10th edition
IQR: Interquartile range
SMD: Standardized mean differences
MOR: Median odds ratio
MI: Myocardial infarction
OR: Odds ratio
HR: Hazard ratio
CI: Confidence interval
SES: Socio-economic status
