# Supplementary material for: Healthy lifestyle behaviours and all-cause and cardiovascular mortality among 0.9 million Chinese adults
Source: Int J Behav Nutr Phys Act. 2021 Dec 18;18:162. doi: 10.1186/s12966-021-01234-4 (PMC8684211; doi:10.1186/s12966-021-01234-4)
Supplement: Supplementary file 1 — Additional file 1. Supplementary material and tables. [file 12966_2021_1234_MOESM1_ESM.docx]

**SUPPLEMENTARY**

**Section 1. Detailed information about sampling selection of China Patient-centered Evaluative Assessment of Cardiac Events (PEACE) Million Persons Project (MPP)**

From September 2015 to November 2019, we selected 252 sites (152 rural counties, 100 urban districts) from all 31 provinces based on their geographic locations within each province, the number of residents living in rural or urban areas, minority ethnicity distribution, quality of disease and death registries, and local capacity to support the project. Specifically, the staff in provincial coordinating office collected basic information (geographic information, economic development, population size, and minority ethnicity distribution) about the selected sites in their province; reported the information to the national coordinating office; and discussed with staff in the national coordinating office to determine the study sites. In each site, about 8 towns or sub-districts were chosen according to their population size, population stability (e.g., no sudden significant change in the number of residents), local staff’s commitment, and ability to perform the screening. Initial screening stations were set up in each town or sub-district health centre.

Potentially eligible participants were identified in each town or sub-district through official residential records and then invited by local community workers via telephone or extensive publicity campaigns on television and in newspaper. All participants were required to bring their identity cards to the screening clinics to verify that they met the inclusion criteria: 1) aged 35 to 75 years; 2) registered in Hukou of the selected site (a record officially identifying a person as a resident of an area), and lived in the selected regions at least 6 months during the last 12 months. After the verification, eligible participants who had signed the informed consent agreement were then enrolled in the project.

**Figure S1. The distribution of project sites in seven regions of mainland China in China Patient-centered Evaluative Assessment of Cardiac Events (PEACE) Million Persons Project (MPP)**


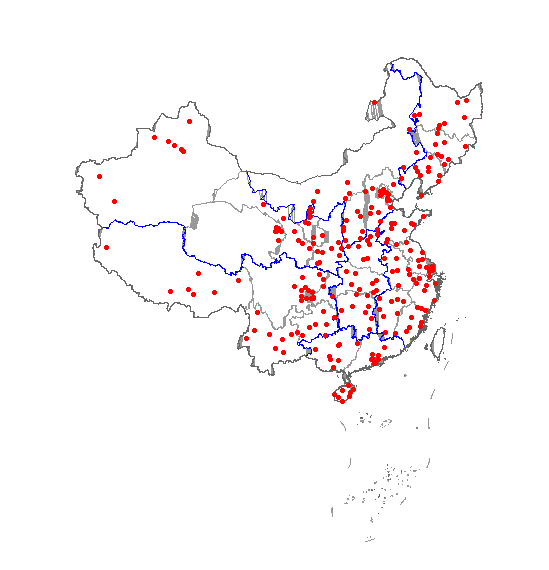


**Provinces/autonomous region/municipality in 7 regions of Mainland China:**

North China: Beijing, Tianjin, Hebei, Shanxi, Inner Mongolia

Northeast: Liaoning, Jilin, Heilongjiang

Central China: Henan, Hubei, Hunan

East China: Shanghai, Jiangsu, Zhejiang, Anhui, Fujian, Jiangxi, Shandong

Northwest: Shaanxi, Gansu, Qinghai, Ningxia, Xinjiang

South China: Guangdong, Guangxi, Hainan

Southwest: Chongqing, Sichuan, Guizhou, Yunnan, Tibet

**Figure S2. Study participants**


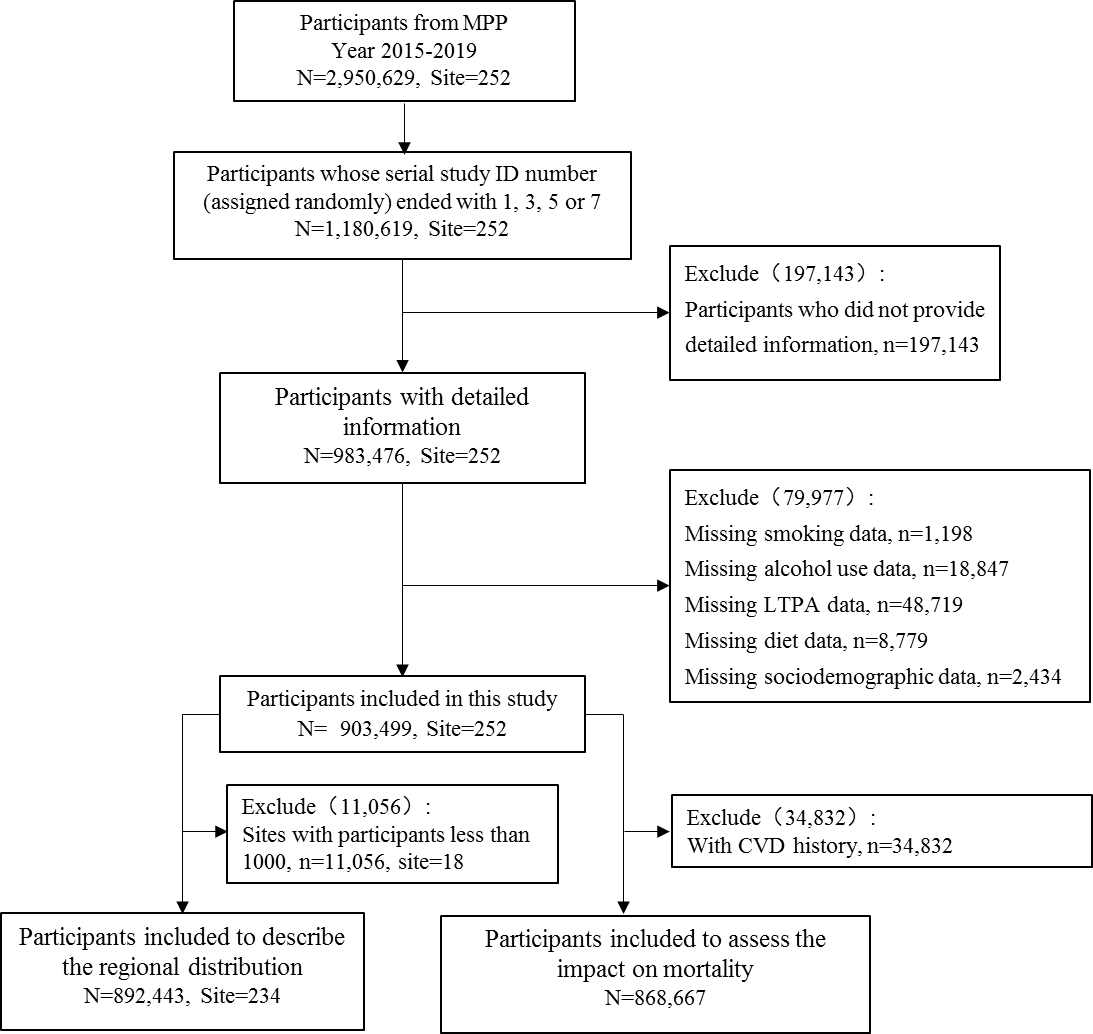


* MPP: China Patient-centered Evaluative Assessment of Cardiac Events Million Persons Project;

LTPA: leisure time physical activity;

CVD: cardiovascular disease

**Table S1. Basic characteristics among participants, group by whether provided lifestyle information**

|  | **Study ID ending with 2, 4, 6, 8, 9, 0** (not required to provide lifestyle information) | **Serial study ID number ending with 1, 3, 5, or 7** | |
| --- | --- | --- | --- |
|  |  | Provided lifestyle information | Required but not provided lifestyle information |
|  | N= 1,770,010 | N=983,476 | N= 197,143 |
| Female | 1062059 (60.0) | 590779 (60.1) | 118112 (59.9) |
| Age, year | 55.8±10 | 55.9±9.9 | 55.1±10.2 |
| 35-39 | 101943 (5.8) | 54155 (5.5) | 13611 (6.9) |
| 40-49 | 414467 (23.4) | 225455 (22.9) | 50724 (25.7) |
| 50-59 | 553951 (31.3) | 310761 (31.6) | 59395 (30.1) |
| 60-69 | 543904 (30.7) | 306612 (31.2) | 56104 (28.5) |
| 70-75 | 155745 (8.8) | 86493 (8.8) | 17309 (8.8) |
| Living in rural area | 1074080 (60.7) | 596360 (60.6) | 119875 (60.8) |
| Occupation: Farmer | 865045 (48.9) | 475785 (48.4) | 100810 (51.1) |
| Education: high school or above | 397213 (22.4) | 223785 (22.8) | 40943 (20.8) |
| Annual household income≥50,000 yuan per year | 299561 (16.9) | 172840 (17.6) | 26957 (13.7) |
| Currently married | 1640782 (92.7) | 913053 (92.8) | 181229 (91.9) |
| Social medical insurance | 1728644 (97.7) | 963896 (98.0) | 189452 (96.1) |

**Section 2. Measurement of healthy lifestyle behaviours**

**Smoking**

Participants were inquired about smoking status (never, former, or current smokers). Ever smokers were further asked about the frequency and type of cigarette smoking as well as the amount of tobacco consumed per day. Former smokers were additionally asked about the reasons for cessation.

**Alcohol consumption**

For alcohol consumption, habitual drinking frequency (‘never’, ‘once or less per month’, ‘2-4 times per month’, ‘2-3 times per week’, ‘more than 4 times per week’) was asked in the questionnaire. For ever drinkers, the amount of alcohol consumption during a typical drinking day was further asked using an ordinal variable, including ‘1 or 2 units’, ‘3 or 4 units’, ‘5 or 6 units’, ‘7 to 9 units’ and ‘10 units or above’. One unit in the evaluation of alcohol consumption is equal to pure alcohol 17ml (≈14g), which equals to about wine 120mL, beer 360mL, or liquor 45mL. An average daily alcohol consumption was estimated on the basis of these information.

**Estimation of average daily alcohol consumption**

| **Drinking frequency** | **Values** | **Amount of alcohol consumption during a typical drinking day** | **Values** |
| --- | --- | --- | --- |
| never | 0 | 1 or 2 units | 1.5 |
| once or less per month | 0 | 3 or 4 units | 3.5 |
| 2-4 times per month | 0 | 5 or 6 units | 5.5 |
| 2-3 times per week | 2.5 | 7 to 9 units | 8.0 |
| more than 4 times per week | 5.5 | 10 units or above | 10.0 |

**Leisure time physical activity (LTPA)**

We measured habitual exercise by asking about typical type of activity at different intensity levels (vigorous or moderate), frequency, and exercise time per week.

**Typical type and intensity of LTPA**

| **Type** | **Intensity** |
| --- | --- |
| Tai-Chi/qigong/leisure walking | Moderate |
| Running/aerobic exercise | Vigorous |
| Ball games (e.g., basketball, table tennis, badminton) | Moderate |
| Brisk walking/gymnastics/folk dancing | Moderate |
| Swimming | Vigorous |
| Other exercise (e.g., mountain walking, home exercise and rope jumping) | Moderate |

* LTPA: leisure time physical activity

**Assessment of food intake**

Habitual food intake frequency during the past year was asked in the questionnaire: ‘daily’, ‘4-6 days per week’, ‘1-3 days per week’, ‘1-3 days per month’, ‘never or almost never’. In this study we focused on 6 food groups: fresh fruit, fresh vegetable, whole grains (mainly referring to crops except rice and flour, including millet, corn, sorghum, sweet potato, etc.), fish and other seafood (referring to fish, shrimp, crab, shellfish, snails, etc.), bean and bean products (referring to all kinds of soy foods including tofu, dried tofu, beverage made from soybeans, etc.), and red meat (referring to a variety of fresh or processed meat such as pork, beef, lamb, etc.).

**The definition of healthy diet score**

| **Food groups** | **‘Healthy’ (score=1)** |
| --- | --- |
| Fresh fruit | every day per week |
| Fresh vegetables | every day per week |
| Whole grains | every day per week |
| Fish and other seafood | ≥ 1 day per week |
| Bean and bean products | ≥ 4 days per week |
| Red meat | <7 days per week |

**Table S2. Clustering of four healthy lifestyle behaviours**

| **Lifestyle behaviours** | **Non-smoking** | **None or moderate alcohol use** | **Sufficient LTPA** | **Healthy diet** | **Adherence** | | |
| --- | --- | --- | --- | --- | --- | --- | --- |
|  |  |  |  |  | **Observed % (95%CI)** | **Expected %** | **O/E** |
| 4 | **+** | **+** | **+** | **+** | 3.40 (3.36, 3.44) | 2.26 | 1.51 |
| 3 | + | + | + | - | 17.59 (17.51, 17.67) | 17.94 | 0.98 |
|  | + | + | - | + | 5.73 (5.68, 5.77) | 6.31 | 0.91 |
|  | + | - | + | + | 0.06 (0.05, 0.06) | 0.08 | 0.76 |
|  | - | + | + | + | 0.63 (0.61, 0.64) | 0.59 | 1.06 |
| 2 | + | + | - | - | 51.34 (51.23, 51.44) | 50.14 | 1.02 |
|  | + | - | + | - | 0.30 (0.29, 0.31) | 0.62 | 0.48 |
|  | + | - | - | + | 0.06 (0.06, 0.07) | 0.22 | 0.29 |
|  | - | + | + | - | 3.92 (3.88, 3.96) | 4.68 | 0.84 |
|  | - | + | - | + | 1.13 (1.11, 1.15) | 1.65 | 0.69 |
|  | - | - | + | + | 0.06 (0.05, 0.06) | 0.02 | 2.88 |
| 1 | + | - | - | - | 0.84 (0.82, 0.86) | 1.74 | 0.48 |
|  | - | + | - | - | 12.91 (12.84, 12.98) | 13.08 | 0.99 |
|  | - | - | + | - | 0.40 (0.39, 0.41) | 0.16 | 2.46 |
|  | - | - | - | + | 0.11 (0.10, 0.12) | 0.06 | 1.95 |
| 0 | - | - | - | - | 1.53 (1.50, 1.55) | 0.45 | 3.36 |

* LTPA: leisure time physical activity;

Observed: observed adherence, identified in the study population;

Expected: expected adherence, computed by multiplying the separate probabilities of each lifestyle on the basis of their occurrence;

O/E: the ratio between the observed and expected adherence; the clustering was identified where O/E ratio was above 1;

**Figure S3. Participants’ adherence to four healthy lifestyle behaviours, by gender and by urbanicity**

**
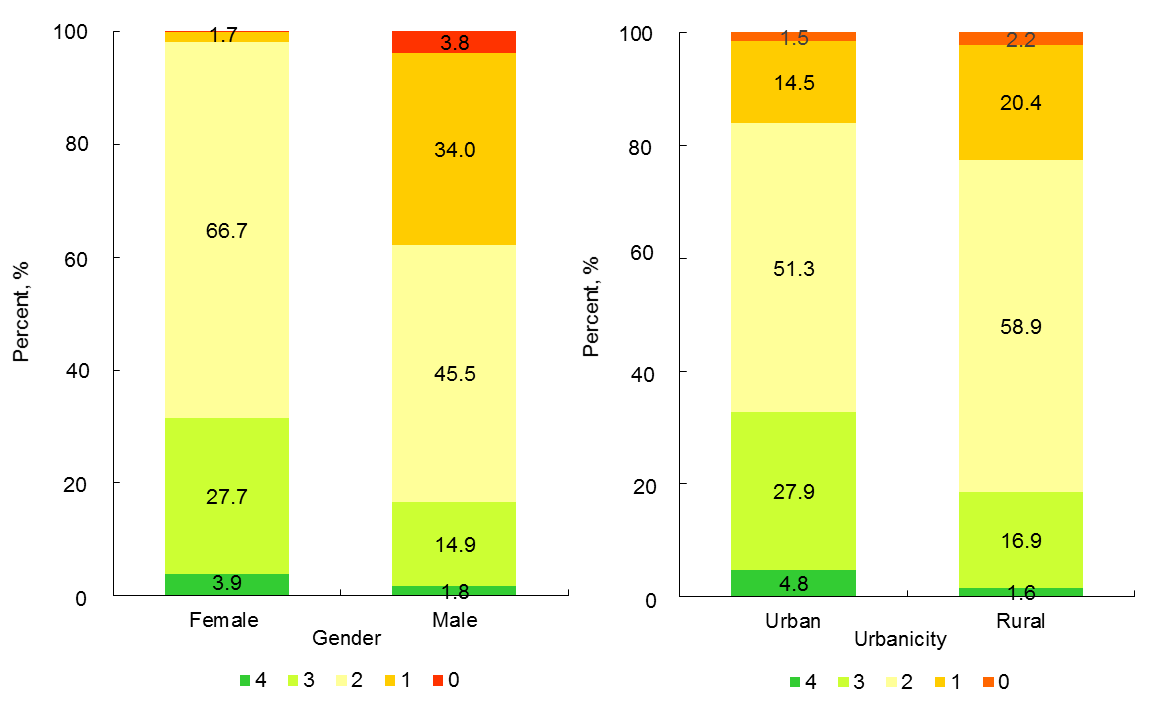
**

* The adherence to each particular number of healthy lifestyle behaviours was rounded to the nearest tenth, and the cumulative percentage for some categories was not precisely 100;

All percents were standardized by age and gender using the 2010 national census data

**Table S3. Standardized county-level adherences to all four healthy lifestyle behaviours among seven regions in mainland China**

|  | **No. counties** | **Median, %** | **Maximum, %** | **Minimum, %** |
| --- | --- | --- | --- | --- |
| Overall | 234 | 1.816 | 14.898 | 0.000 |
| Central | 24 | 1.610 | 9.776 | 0.032 |
| East | 56 | 1.625 | 11.881 | 0.000 |
| North | 42 | 3.334 | 14.898 | 0.125 |
| Northeast | 22 | 2.375 | 9.407 | 0.135 |
| Northwest | 34 | 1.947 | 9.964 | 0.000 |
| South | 21 | 1.584 | 5.420 | 0.096 |
| Southwest | 35 | 0.775 | 9.030 | 0.000 |

* All percentages were standardized by age and gender using the 2010 national census data

**Table S4. Standardized adherence to healthy lifestyle behaviours among seven regions**

|  |  | **Non-smoking** | **MALC** | **Sufficient LTPA** | **Healthy diet** | **ALL** |
| --- | --- | --- | --- | --- | --- | --- |
| Overall | 903499 | 74.1 (74.1, 74.2) | 96.0 (96.0, 96.0) | 23.6 (23.5, 23.7) | 11.1 (11.0, 11.2) | 2.8 (2.8, 2.9) |
| Central | 117158 | 72.8 (72.6, 73.1) | 95.6 (95.5, 95.7) | 25.0 (24.7, 25.2) | 8.8 (8.6, 8.9) | 2.7 (2.6, 2.8) |
| East | 233254 | 75.9 (75.7, 76.1) | 94.3 (94.3, 94.4) | 20.9 (20.7, 21.1) | 11.1 (10.9, 11.2) | 3.0 (2.9, 3.1) |
| North | 170244 | 72.8 (72.6, 73.0) | 95.6 (95.5, 95.7) | 26.9 (26.7, 27.1) | 15.9 (15.7, 16.1) | 3.9 (3.8, 4.0) |
| Northeast | 74629 | 77.0 (76.7, 77.3) | 97.0 (96.8, 97.1) | 19.6 (19.3, 19.8) | 15.6 (15.4, 15.9) | 2.8 (2.7, 2.9) |
| Northwest | 115709 | 72.7 (72.4, 72.9) | 98.5 (98.4, 98.6) | 27.2 (26.9, 27.4) | 10.1 (9.9, 10.3) | 2.6 (2.6, 2.7) |
| South | 60123 | 76.8 (76.5, 77.1) | 97.1 (97.0, 97.3) | 25.9 (25.5, 26.2) | 7.7 (7.5, 7.9) | 2.2 (2.1, 2.3) |
| Southwest | 132382 | 72.5 (72.2, 72.7) | 96.6 (96.5, 96.7) | 21.2 (21.0, 21.5) | 6.9 (6.7, 7.0) | 1.8 (1.7, 1.9) |
| MOR |  | 1.6 (1.6, 1.7) | 2.2 (2.1, 2.4) | 2.6 (2.4, 2.8) | 2.7 (2.5, 3.0) | 3.4 (3.0, 3.8) |
| P value |  | 0.0008 | <0.0001 | 0.2292 | <0.0001 | 0.0167 |

* All percentages were standardized by age and gender using the 2010 national census data;

MALC: none or moderate alcohol use;

LTPA: leisure time physical activity;

ALL: all four healthy lifestyle behaviours;

MOR: median odds ratio;

P values: P values for KW test

**Figure S4. Correlations of the adherence to all four healthy lifestyle behaviours with per capital gross domestic product (GDP) and average annual temperature across counties or districts**

**
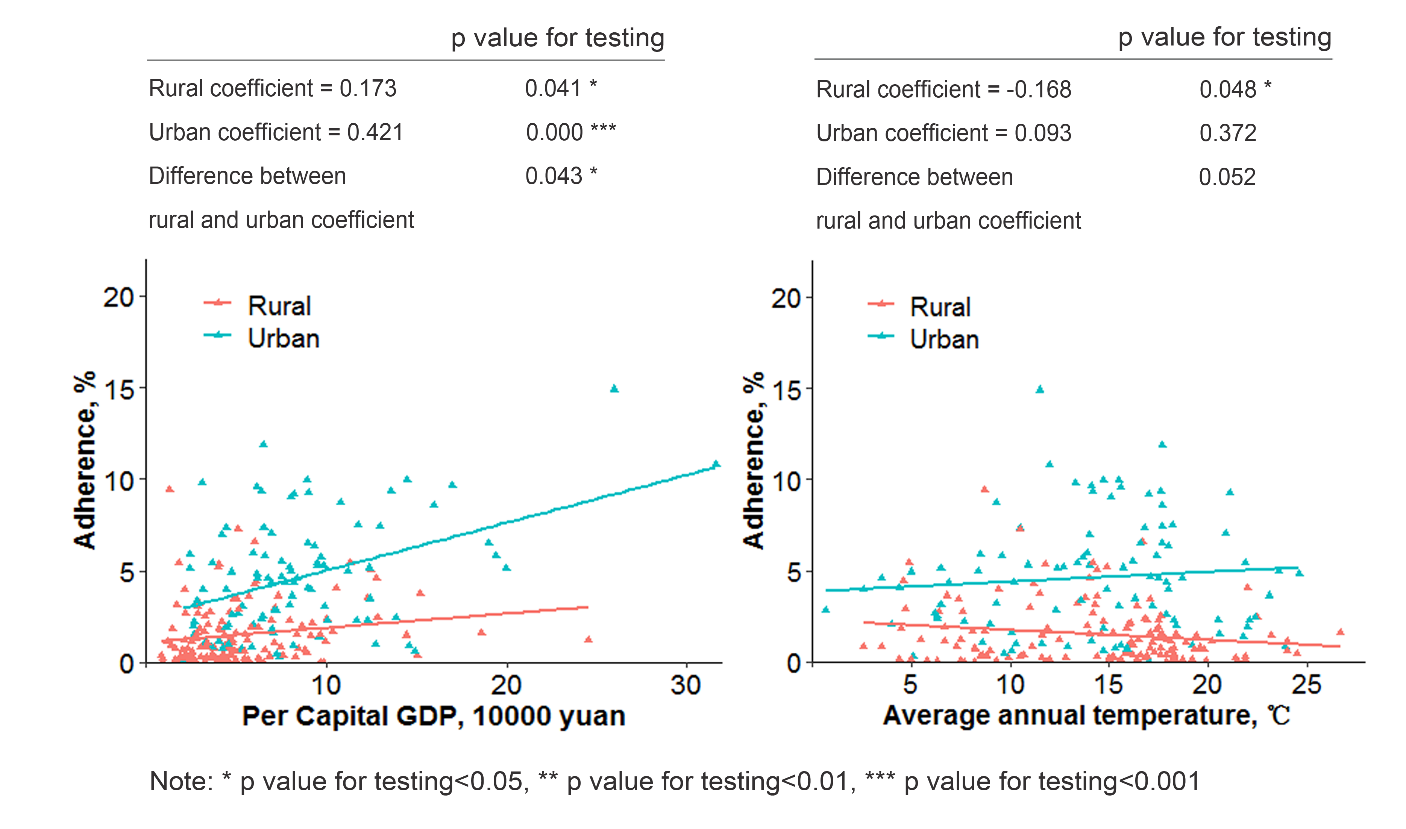
**

* GDP: gross domestic product

**Figure S5. Log-minus-log plots for all-cause and cardiovascular mortality**

**
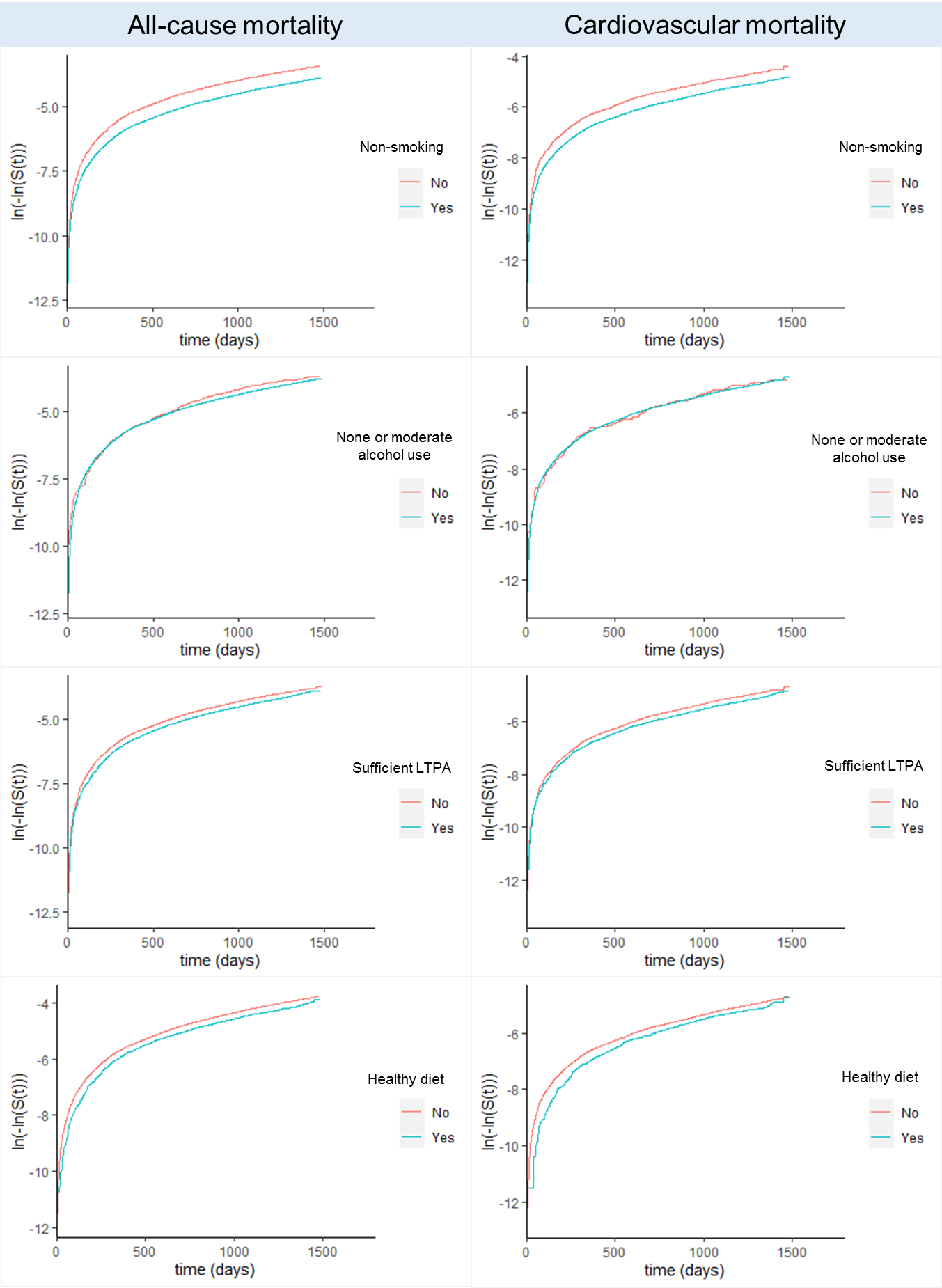
**

* LTPA: leisure time physical activity

**Table S5. Hazard ratios for all-cause and cardiovascular mortality when excluding the deaths in the first six months (N=** **867,535)**

|  | **All-cause mortality** | |  | **Cardiovascular mortality** | |
| --- | --- | --- | --- | --- | --- |
|  | **HR (95% CI)** | **P value** |  | **HR (95% CI)** | **P value** |
| Number of healthy lifestyle behaviours |  |  |  |  |  |
| 0 | Reference |  |  | Reference |  |
| 1 | 0.94 (0.80, 1.10) | 0.445 |  | 0.97 (0.74, 1.28) | 0.847 |
| 2 | 0.88 (0.75, 1.03) | 0.102 |  | 0.90 (0.69, 1.18) | 0.457 |
| 3 | 0.73 (0.62, 0.86) | <0.001 |  | 0.75 (0.56, 1.00) | 0.047 |
| 4 | 0.64 (0.52, 0.81) | <0.001 |  | 0.55 (0.37, 0.81) | 0.003 |
| Non-smoking | 0.87 (0.82, 0.92) | <0.001 |  | 0.90 (0.81, 0.99) | 0.039 |
| None or moderate alcohol use | 1.04 (0.93, 1.17) | 0.485 |  | 1.02 (0.83, 1.24) | 0.874 |
| Sufficient LTPA | 0.83 (0.78, 0.88) | <0.001 |  | 0.77 (0.70, 0.85) | <0.001 |
| Healthy diet | 0.91 (0.84, 0.99) | 0.036 |  | 0.92 (0.80, 1.05) | 0.231 |

* LTPA: leisure time physical activity;

All models were adjusted for gender, age, occupation, education, household income, marriage, social medical insurance, urbanicity, region, and county level per capital gross domestic product (GDP)

**Table S6. Hazard ratios for all-cause and cardiovascular mortality when excluding the persons who had self-reported hypertension or diabetes (N=** **636,000)**

|  | **All-cause mortality** | |  | **Cardiovascular mortality** | |
| --- | --- | --- | --- | --- | --- |
|  | **HR (95% CI)** | **P value** |  | **HR (95% CI)** | **P value** |
| Number of healthy lifestyle behaviours |  |  |  |  |  |
| 0 | Reference |  |  | Reference |  |
| 1 | 0.89 (0.75, 1.06) | 0.208 |  | 0.88 (0.64, 1.21) | 0.428 |
| 2 | 0.86 (0.73, 1.03) | 0.097 |  | 0.82 (0.60, 1.12) | 0.211 |
| 3 | 0.70 (0.58, 0.85) | <0.001 |  | 0.69 (0.49, 0.97) | 0.030 |
| 4 | 0.66 (0.51, 0.86) | 0.002 |  | 0.45 (0.27, 0.75) | 0.002 |
| Non-smoking | 0.91 (0.85, 0.97) | 0.008 |  | 0.94 (0.83, 1.06) | 0.326 |
| None or moderate alcohol use | 1.09 (0.95, 1.25) | 0.231 |  | 0.96 (0.75, 1.23) | 0.748 |
| Sufficient LTPA | 0.80 (0.74, 0.86) | <0.001 |  | 0.74 (0.65, 0.84) | <0.001 |
| Healthy diet | 0.91 (0.82, 1.01) | 0.063 |  | 0.90 (0.76, 1.07) | 0.226 |

* LTPA: leisure time physical activity;

All models were adjusted for gender, age, occupation, education, household income, marriage, social medical insurance, urbanicity, region, and county level per capital gross domestic product (GDP)

**Table S7. Hazard ratios (HRs) of none or moderate alcohol use for all-cause and cardiovascular mortality based on two different variable definitions**

|  | **All-cause mortality** | |  | **Cardiovascular mortality** | |
| --- | --- | --- | --- | --- | --- |
|  | **HR (95% CI)** | **P value** |  | **HR (95% CI)** | **P value** |
| **Model 1: no adjustment** | | | | | |
| Chinese dietary guideline | 0.88 (0.79, 0.98) | 0.019 |  | 0.98 (0.82, 1.18) | 0.855 |
| US dietary guideline | 0.84 (0.75, 0.93) | 0.001 |  | 0.97 (0.81, 1.17) | 0.777 |
| **Model 2: adjusted for age and gender** | | | | | |
| Chinese dietary guideline | 1.07 (0.96, 1.19) | 0.214 |  | 1.13 (0.94, 1.35) | 0.184 |
| US dietary guideline | 1.06 (0.95, 1.18) | 0.325 |  | 1.17 (0.97, 1.41) | 0.102 |
| **Model 3: multivariable adjusted model** | | | | | |
| Chinese dietary guideline | 1.08 (0.97, 1.21) | 0.156 |  | 1.05 (0.87, 1.27) | 0.587 |
| US dietary guideline | 1.07 (0.95, 1.20) | 0.246 |  | 1.09 (0.89, 1.32) | 0.398 |

* Two definitions for “none or moderate alcohol use” :

a. based on Chinese dietary guideline: never drinkers, or drinkers who drank no more than 25g (for male) or 15g (for female) per day on average;

b. based on US dietary guideline: never drinkers, or drinkers who drank no more than 14 units (for male) or 7 units (for female) per week on average
